# Mobile application for self-care in people with heart failure: a quasi-experimental study*

**DOI:** 10.1590/1980-220X-REEUSP-2025-0537en

**Published:** 2026-09-11

**Authors:** Wallison Pereira dos Santos, Mailson Marques de Sousa, André Atanasio Maranhão Almeida, Taciana da Costa Farias Almeida, Fernanda Beatriz Dantas de Freitas, Bernadete de Lourdes André Gouveia, Simone Helena dos Santos Oliveira

**Affiliations:** 1 Universidade Federal de Campina Grande, Centro de Formação de Professores, Unidade Acadêmica de Enfermagem, Cajazeiras, PB, Brazil.; 2 Universidade Federal da Paraíba, Centro de Ciências da Saúde, Departamento de Enfermagem Clínica, João Pessoa, PB, Brazil.; 3 Instituto Federal de Educação, Ciência e Tecnologia da Paraíba, Esperança, PB, Brazil.; 4 Universidade Federal de Campina Grande, Centro de Ciências Biológicas e da Saúde, Unidade Acadêmica de Enfermagem, Campina Grande, PB, Brazil.; 5 Hospital de Emergência e Trauma Senador Humberto Lucena, Departamento de Enfermagem, João Pessoa, PB, Brazil.; 6 Universidade Federal de Campina Grande, Centro de Educação e Saúde, Unidade Acadêmica de Enfermagem, Cuité, PB, Brazil.; 7 Universidade Federal da Paraíba, Centro de Ciências da Saúde, Escola Técnica de Saúde, João Pessoa, PB, Brazil.

**Keywords:** Heart Failure, Self Care, Mobile Applications, Biomedical Technology, Nursing

## Abstract

**Objective:**

To compare the self-care scores of people with heart failure before and after using the “Tum-Tum” mobile application.

**Method:**

A single-group, quasi-experimental, before-and-after study involving a sample of 57 individuals treated at the cardiology outpatient clinic of a public university hospital in the city of João Pessoa, Paraíba, conducted between January and May 2024. To measure self-care, the Self-Care of Heart Failure Index was used before (T0) and 60 days after (T1) using the mobile application. Data analysis involved descriptive statistics and the Wilcoxon signed-rank test.

**Results:**

54.4% of participants were male. The median age was 60 years, and the median number of years of education was six years. A statistically significant difference was observed between the pre-intervention (T0) and post-intervention (T1) time points regarding the heart failure self-care maintenance and self-care confidence subscales.

**Conclusion:**

The use of the “Tum-Tum” mobile app had a positive influence on increasing scores. The app has the potential to support educational strategies promoted by healthcare professionals—particularly nurses—regarding self-care for people with heart failure. The study was registered with ReBEC – registry RBR-5zx8rvc.

## INTRODUCTION

Self-care plays an important role in heart failure (HF) management. As this syndrome is characterized by structural and functional changes in the heart—resulting in an inability to pump an adequate volume of blood—adherence to an effective therapeutic regimen combining pharmacological and non-pharmacological treatments is necessary to slow the syndrome’s progression and maintain functional capacity and health-related quality of life^(1)^.

Non-pharmacological treatment includes measures that reflect self-care by the individual with HF, such as daily body weight monitoring, regular physical activity, fluid restriction, a low-sodium diet, smoking and alcohol cessation, and keeping vaccinations up to date^(2)^.

Educational interventions can improve self-care behaviors by raising awareness and fostering an individual’s engagement with their clinical condition. Nurse-led health education can result in significant changes in an individual’s lifestyle. It is understood that nursing interventions must be grounded in solid scientific evidence and a theoretical framework that guides clinical practice, with the aim of promoting safe and comprehensive care for people with HF^(3,4)^.

The Situation-Specific Theory of HF Self-Care conceptualizes self-care as a naturalistic decision-making process involving the selection of behaviors to maintain physiological stability (self-care maintenance) and to respond to symptoms when they occur. Therefore, self-care behavior encompasses a variety of actions that reflect active engagement and responsibility for self-care maintenance, self-care monitoring, and self-care management, as well as the confidence to perform these activities^(5)^. In this context, interventions based on theoretical models to promote self-care behaviors are timely and necessary.

In HF, the use of technological resources has emerged as an expanding tool. Mobile applications (apps) have been used as an adjunctive tool for interventions aimed at promoting self-care, monitoring signs and symptoms, and supporting disease control and therapeutic management. Among the benefits of their use are the absence of time and geographical constraints. However, limitations related to internet access and adequate health literacy for the use of these technologies still persist^(6)^.

The “Tum-Tum” app, developed in the Brazilian context, demonstrates adequate evidence of content, face, and usability validity, featuring self-explanatory interfaces that cover concepts regarding the syndrome, main causes, signs and symptoms, diagnosis, treatment, and self-care measures for people with HF. Content validity was conducted by a panel of six expert nurses and resulted in an overall Content Validity Index of 88.4%. Face validity assessment with the target audience covered the app’s organization, illustrations, figures, font size, content, language, and motivation for use^(6)^. The usability assessment yielded satisfactory scores, with participants agreeing on clarity, language, ease of use, and utility, in addition to indicating a willingness to recommend the app to other users^(7)^.

“Tum-Tum” provides features for monitoring signs and symptoms of clinical decompensation, recording daily body weight, and setting reminders for current medications, medical tests, and scheduled appointments. The app can be used without an internet connection, making it convenient to use anywhere and at any time^(6,7)^.

It is pertinent to assess the potential effects and implications of using health technologies to promote self-care among the Brazilian population with HF, taking into account high morbidity and mortality rates, hospital bed occupancy rates, the financial burden on public funds, and the impact on the quality of life of those living with the disease^(8)^. From this perspective, the results of this research may facilitate the introduction and implementation of educational strategies to support the care of this population. This study aimed to compare the self-care scores of people with HF measured before and after using the “Tum-Tum” mobile application.

## METHOD

### Study Design

This is a single-group, quasi-experimental, before-and-after study, structured in accordance with the Transparent Reporting of Evaluations with Nonrandomized Designs recommendations.

### Study Setting

The study was conducted at the cardiology outpatient clinic of a public university hospital in the city of João Pessoa, Paraíba, Brazil.

### Eligibility Criteria

Eligible participants were those with a clinical diagnosis of chronic HF who were receiving outpatient follow-up care and demonstrated adequate health literacy (HL) (≥19 points), as assessed using the Eight-Item Health Literacy Scale^(9)^. This scale assesses the ability to obtain, process, and understand basic health information needed to make appropriate health decisions. Restricting eligibility to individuals with adequate HL was intended to ensure the autonomous use of the application, without the need for assistance from third parties, thereby minimizing external influences that could compromise the reliability of the recorded information.

### Population and Sample

The study population consisted of individuals with HF receiving follow-up care. The sample size was calculated using the public-domain software “Sample Size Calculation”, based on a 5% margin of error, a 95% confidence level, and an estimated population standard deviation of 15.7, identified in a previous study involving Brazilian patients^(10)^. A minimum sample of 41 individuals with HF was required.

Individuals with chronic HF aged ≥18 years who owned a smartphone with the Android operating system, were receiving pharmacological treatment, and were oriented to time and place were included in the study. Orientation to time and place was assessed by the principal investigator using the following questions: what is your name? How old are you? What are today’s day, month, year, and where are we now? What city do you live in?^(11)^.

Individuals were excluded if, due to clinical decompensation, they had been hospitalized within the previous 30 days, were on the heart transplant waiting list, and/or were scheduled to undergo myocardial revascularization or a transcatheter aortic valve implantation procedure, as these patients frequently experience clinical instability, which would make the data collection phase unfeasible. During the course of the study, it was feasible to recruit a final sample of 57 participants.

### Data Collection Period and Strategy

The study was conducted between January and May 2024. Individuals with HF were approached in person in the waiting room on the day of their scheduled outpatient medical appointment. The application was downloaded onto the participant’s smartphone, after which they were introduced to its interface and functionalities, including setting reminders for prescribed medications, medical appointments, and recording signs and symptoms. Participants also received verbal guidance on the importance of adopting self-care behaviors for the clinical management of HF, which are also addressed within the application. It should be noted that the link to access the application was sent via WhatsApp^®^, and the participant’s telephone number was recorded for subsequent contact.

Subsequently, participants navigated through the app and were informed that they would receive two follow-up phone calls—15 and 30 days after downloading—lasting approximately 10 minutes each, aimed at identifying questions and difficulties related exclusively to the tool’s operation. Data regarding clinical condition were not investigated. Contacts were conducted using a semi-structured script comprising the following questions: Do you experience any difficulty accessing the app? If so, what kind? How many times have you used the app in the last 15/30 days? Which features have met your needs? Do you see a need for interface improvements? Do you have any suggestions?

**Figure 1:**
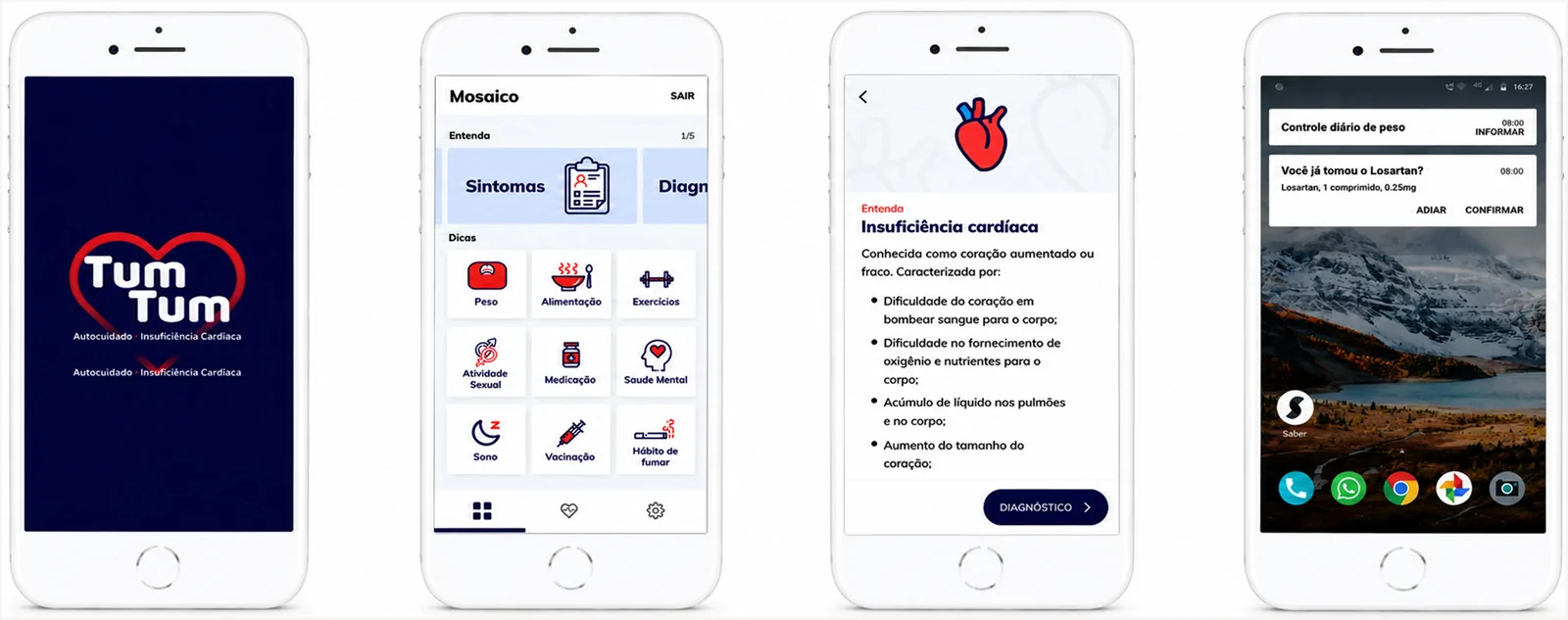
Screens from the “Tum-Tum” mobile app – João Pessoa, PB, Brazil, 2024.

Source: “Tum-Tum” interface, 2024.

The intervention consisted of using the “Tum-Tum” mobile app for eight weeks following recruitment (T0), concluding 60 days after app usage (T1). It is worth noting that the 60-day period is considered a likely timeframe for the occurrence of behavioral changes regarding health self-care^(12)^. “Tum-Tum” is an app prototype designed to provide educational information focused on fostering self-care in HF by promoting knowledge. The app contains information about HF—including causes, symptoms, diagnosis, and treatment—organized into educational topics covering weight, diet, exercise, sexual activity, medications, mental health, sleep, vaccination, and smoking habits^(6,7)^. The app screens are shown in Figure 1.

A form containing questions regarding the conditions of individuals with HF—previously used in a prior study—was employed to collect sociodemographic and clinical information^(6)^.

The level of self-care was measured in person at the outpatient clinic using the Brazilian version of the Self-Care of Heart Failure Index (SCHFI)^(13,14)^ at two time points: pre-intervention (T0) and post-intervention (T1) with the mobile application. The SCHFI assesses self-care behaviors using 22 items distributed across three subscales: maintenance (ten items), management (six items), and self-care confidence (six items). Each subscale should be analyzed independently, with scores ≥ 70 points indicating adequate self-care^(13,14)^.

### Data Analysis

For the statistical analyses, data were compiled and then processed using the statistical software R. Descriptive statistics were employed, presenting simple absolute frequencies and percentages for the categorical variables. Subsequently, the chi-square goodness-of-fit test was applied to verify the suitability of the probabilistic model to the survey data.

The data were not normally distributed. Numerical variables were described using the median and interquartile range (first quartile–third quartile). A violin plot, also known as a box-and-whisker plot, is presented to enable visualization of the paired data at T0 and T1, showing the distribution of participants across this interval, as well as the mean values, first quartile, median, and third quartile.

The analysis of self-care using the SCHFI is based on measuring the scores obtained individually for each subscale (maintenance, management, and confidence), with values ≥ 70 points indicating adequate self-care. To compare potential differences in self-care scores following app usage—specifically between the medians at baseline (T0) and after 60 days (T1)—the non-parametric Wilcoxon test, a method for comparing two paired samples, was applied. A significance level of 5% (p-value < 0.05) was adopted for all analyses.

### Ethical Aspects

The study was conducted in accordance with Resolution 466/2012 of the National Health Council of the Brazilian Ministry of Health. It was approved by the Research Ethics Committee of *Hospital Universitário Lauro Wanderley*, *Universidade Federal da Paraíba* (Certificate of Presentation for Ethical Consideration 74304723.1.0000.5183), under Opinion 6,417,699/2023. Moreover, the study was registered with the International Clinical Trials Registry Platform through the Brazilian Registry of Clinical Trials (ReBEC – registration RBR-5zx8rvc).

## RESULTS

A total of 73 potential participants were invited to participate, of whom 13 declined. Of the 60 participants enrolled, three did not complete the follow-up, resulting in a final sample of 57 individuals with HF receiving follow-up care at the cardiology outpatient clinic. Regarding the sociodemographic profile, the median age, years of education, and HL score were 60 years, 6 years, and 27 points, respectively. Most participants were male (n = 31; 54.4%), self-identified as white (n = 19; 33.3%), resided in João Pessoa (n = 39; 68.4%), were married or living in a stable union (n = 32; 56.1%), lived with their partner (n = 29; 50.9%), were retired (n = 27; 47.4%), and had a monthly family income between one and two minimum wages (n = 45; 78.9%).

**Figure 2:**
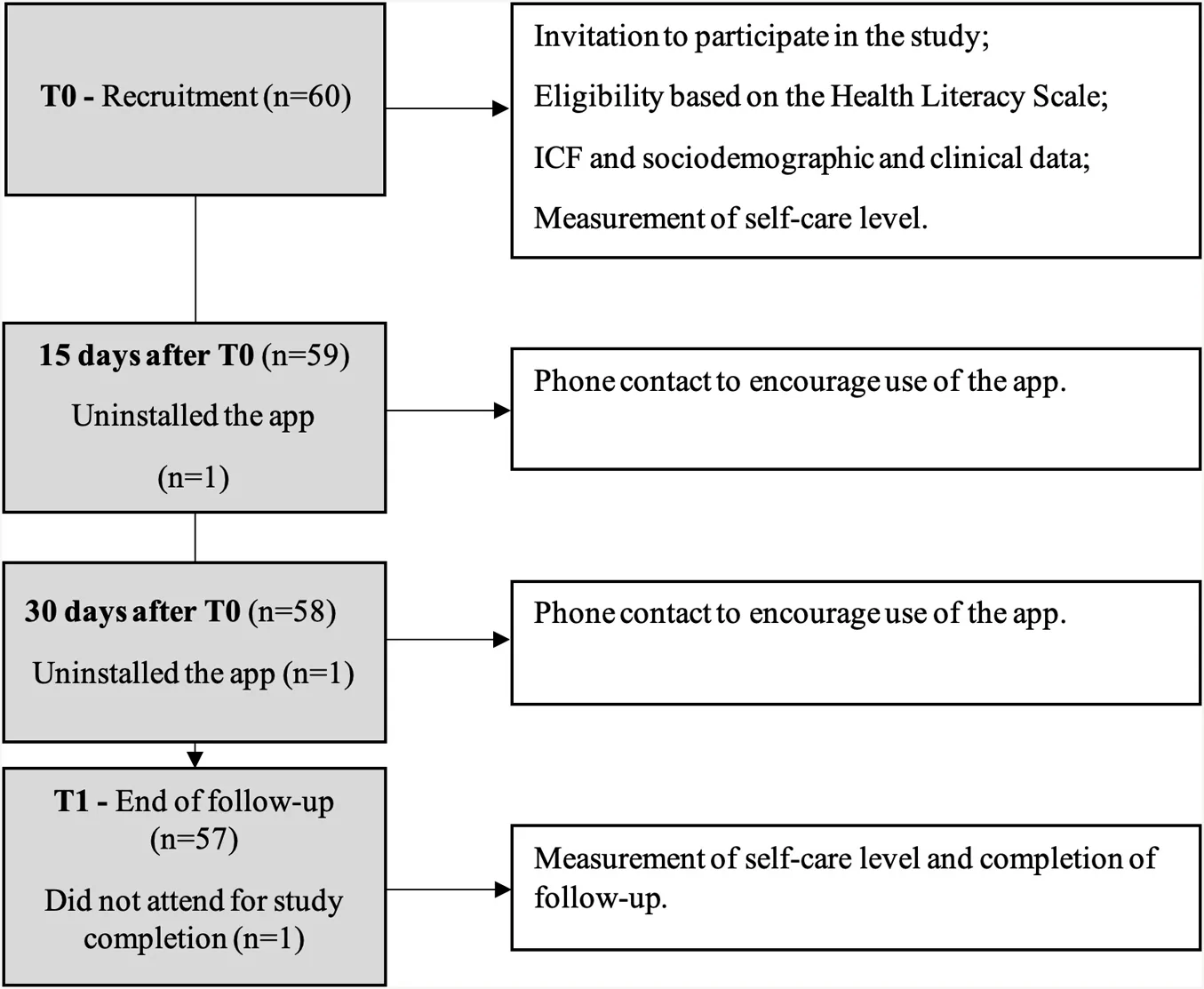
Data collection flowchart – João Pessoa, PB, Brazil, 2024.

Source: Data from collection, 2024.

Most participants had heart failure with reduced left ventricular ejection fraction (n = 35; 61.4%), were classified as New York Heart Association functional class II (n = 31; 54.4%), and had HF of ischemic etiology (n = 32; 56.2%). In terms of HF-related comorbidities, hypertension was the most prevalent (n = 49; 86.0%), and the most frequently used medication class was beta-blockers (atenolol, carvedilol, metoprolol, propranolol, and bisoprolol) (n = 43; 75.4%). Figure 2 presents the data collection flowchart.

Table 1 presents the self-care scores for each subscale at T0 and T1. An increase was observed in the scores of each subscale after the use of the “Tum-Tum” application. A statistically significant difference was observed between T0 and T1 in the self-care maintenance and self-care confidence components.

**Table 1:** Self-care of heart failure index component scores at T0 (pre-intervention) and T1 (post-intervention) – João Pessoa, PB, Brazil, 2024.

Subscales	T0 – Pre-intervention			T1 – Post-intervention		P-value*
	Median	(Q1–Q3)		Median	(Q1–Q3)	
Maintenance	63	(46–76)		66	(63–72)	0.002
Management	59	(49–70)		62	(54–64)	0.063
Confidence	85	(72–81)		95	(72–95)	0.001

Notes: *Wilcoxon signed-rank test.

Source: Survey data (2024).

**Figure 3:**
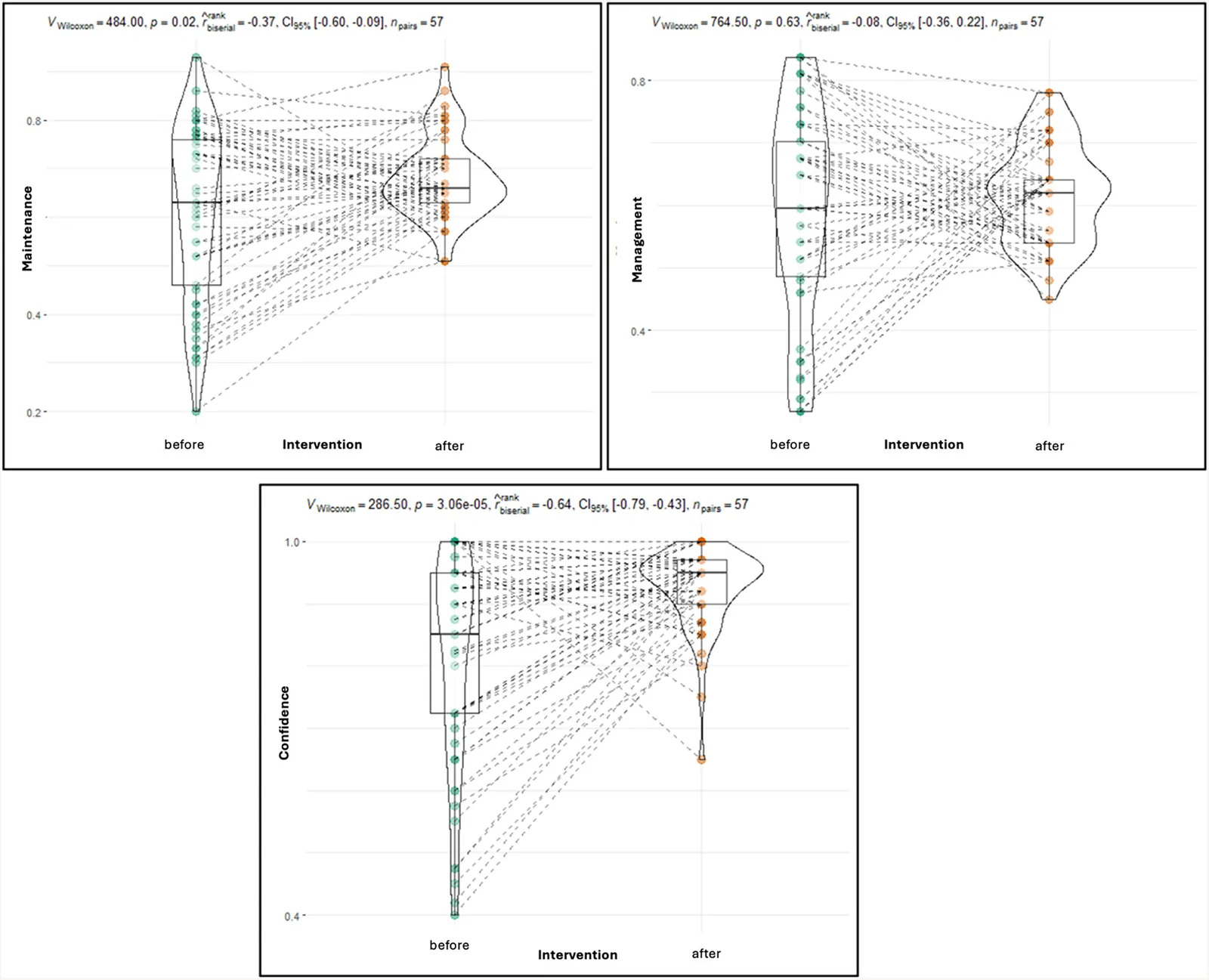
Comparison of self-care maintenance, management, and confidence subscale scores between T0 (pre-intervention) and T1 (post-intervention) – João Pessoa, PB, Brazil, 2024.

Source: Survey data (2024).

When comparing the self-care maintenance subscale scores shown in Figure 3, displayed as a boxplot, it was possible to examine the paired data and the distribution of participants at baseline (T0), with a median score of 63, and after the intervention (T1), with a median score of 66 following the use of the mobile application, demonstrating a statistically significant change. In addition, a shift in the distribution of participants was observed, with a greater concentration of scores above 60 points after the use of the “Tum-Tum” application.

For the self-care management subscale, the scores before the use of the mobile application were more widely dispersed, with values below 40 points (T0). However, after the intervention (T1), the scores became concentrated above 50 points. Although no statistically significant difference was identified, an increase in the self-care management subscale scores was observed.

The comparison of the paired self-care confidence subscale scores between the pre-intervention (T0) and post-intervention (T1) assessments showed that, at pre-intervention, the scores were already adequate, with values ≥70 points. Following the intervention, the scores became concentrated at ≥90 points, demonstrating increased participants’ confidence in performing self-care after using the mobile application, with responses clustered at the upper end of the scale, corresponding to high scores ranging from 90 to 100 points.

The comparison of the scores for each SCHFI subscale between T0 and T1 is presented in Figure 3.

## DISCUSSION

In this study, it was observed that at the initial time point (T0), self-care maintenance and management scores were unsatisfactory. However, at the post-intervention time point (T1), the level of self-care improved—particularly in the maintenance subscale, which reflects behaviors aimed at physiological stability.

Self-care results from the integration of maintenance, management, and confidence behaviors; in other words, the individual must master these components to achieve adequate self-care capable of preventing and mitigating complications arising from clinical decompensation—a state that, in turn, predisposes the individual to frequent readmissions and adverse health outcomes^(5)^.

Maintaining self-care constitutes a proactive practice in which the individual adopts behaviors aimed at preventing complications and preserving physiological stability, encompassing healthy habits and adherence to pharmacological treatment^(5)^. It was observed that, following the use of the “Tum-Tum” mobile app, participants showed a relevant and statistically significant increase in the maintenance subscale, suggesting that they adopted behaviors aimed at maintaining physiological stability after using the app. However, they still remained below the cutoff point considered satisfactory (≥ 70 points).

Self-care management involves a reactive response, in which the individual recognizes signs and symptoms and makes decisions in response to them. In this context, symptom management represents the interface between identifying clinical changes and making appropriate decisions, reflecting a proactive attitude in which the patient selects the self-care behavior most appropriate for their condition^(5)^.

In patients with HF, symptom perception is a challenging task due to the influence of multiple comorbidities that can affect bodily sensations associated with conditions unrelated to HF^(13)^. It can be inferred that people recognize the characteristic signs and symptoms; however, decision-making is limited to the use of oral medications to control the disease’s symptoms—meaning the self-care management component is not fully exercised.

A study conducted in São Paulo supports the previous statement, as all participants showed inadequate scores across the three subscales—to varying degrees—with self-care management standing out as the lowest-scoring area^(10)^. Additionally, a study conducted in Switzerland identified a similar pattern, with self-care management corresponding to the lowest-scoring subscale and confidence reaching adequate scores^(15)^. In contrast, research conducted in Portugal identified the confidence subscale as the one with the lowest score^(16)^.

The Situation-Specific Theory of Heart Failure Self-Care posits that individuals strive to maintain both physical and mental health and, therefore, place their confidence in specific therapeutic interventions^(5)^. At baseline (T0), the self-care confidence subscale already demonstrated satisfactory scores, and after the intervention (T1), an increase in the score was observed while maintaining adequate values. Greater self-care confidence has been shown to be associated with better self-care behaviors^(5)^. This finding is consistent with the results of the present study, in which the increase in the self-care confidence score was accompanied by increases in the self-care maintenance and self-care management scores.

It is important to consider that confidence in self-care directly influences the maintenance and management of self-care, as individuals may place their confidence solely in pharmacological treatment, failing to recognize the need to modify the habits and behaviors required to achieve satisfactory self-care practices.

Previous studies report similar findings, demonstrating unsatisfactory self-care among people with HF^(10,16,17)^. A systematic review with meta-analysis indicated inadequate self-care across all three dimensions of self-care: maintenance, management, and confidence^(18)^. In the Brazilian setting, the findings of the present study at the pre-intervention assessment showed that the self-care maintenance and self-care management subscale scores were below 70 points and were therefore considered inadequate. In contrast, the self-care confidence subscale score exceeded 70 points and was considered adequate^(19)^.

Understanding that the components of self-care both influence and are influenced by external and internal factors ensures that care for the person living with HF is holistic, complex, and comprehensive, while also being individualized—taking into account the social, economic, and cultural context in which the individual is situated^(20)^. Self-care maintenance and management behaviors are directly influenced by the variables of knowledge, lifestyle, and social support^(17,18)^.

Self-care involves individual responsibility, autonomy, and independence, including the ability to engage in one’s own self-care practice^(18)^. It is possible to increase levels of self-care and knowledge through educational strategies utilizing information technologies. A randomized clinical trial—which involved intensive four-day training and cycles every four months over a 12-month period using illustrated educational leaflets—observed an increase in self-care scores in the intervention group compared to the control group^(21)^.

From this perspective, the importance of educational interventions—such as the one developed in this study—for promoting self-care is highlighted. Corroborating the results of this study, research conducted in Japan—which examined the effect of a mobile app on the self-care behavior of patients with chronic HF after two months of use, utilizing the European Heart Failure Self-Care Behaviour Scale—demonstrated an improvement in self-care within the intervention group^(22)^.

In China, a quasi-experimental study observed that the use of a mobile app improved patients’ understanding and awareness of HF regarding self-management skills for the syndrome^(23)^. In South Korea, a pilot study involving a mobile app-based intervention resulted in significant improvements in digital HL scores, disease knowledge, and self-care behaviors among patients with HF^(24)^. Another study demonstrated a significant improvement in self-care quality after three months of using a mobile app^(25)^.

A meta-analysis aimed at assessing the impact of e-health interventions on disease management in patients with HF—including randomized controlled trials from various countries (China, USA, UK, Sweden, Australia)—suggests that information technology-based health interventions are replicable in diverse settings; the primary outcome is an improved prognosis for individuals with the condition, with the potential to reduce mortality and readmissions while fostering an improved quality of life^(26)^.

In the present study, participants showed improvement in scores for self-care maintenance, management, and confidence. The adopted theoretical framework posits that maintenance behaviors are influenced by external factors and that decision-making can occur consciously or unconsciously, based on the interaction between individual characteristics and the context in which an individual is situated^(5)^. It can be inferred, therefore, that exposure to the application had a positive influence on self-care performance, with the potential to be incorporated as a support tool in educational strategies conducted by nurses and the multidisciplinary team.

Among the limitations of this study, notable aspects include the restricted profile of participants with adequate HL levels, as well as the multifactorial nature of self-care, which is influenced by conditions imposed by the syndrome itself, advanced age, low educational attainment, psychological factors, and insufficient social support^(1,5)^. These characteristics limit the generalizability of the findings, which should be interpreted with caution. Additionally, self-reported app usage is subject to social desirability bias, and the absence of objective metrics regarding engagement, access, and usage time represents a further limitation to be addressed in future research. It is recommended to conduct randomized clinical trials using the “Tum-Tum” app—with larger samples, longer follow-up periods, and implementation across different care settings—to enhance the comparability of findings and assess the tool’s efficacy.

## CONCLUSION

This study identified an increase in the maintenance, management and self-care confidence scores of people with cardiac failure after using the “Tum-Tum” mobile application. The results provide healthcare professionals—especially nurses—with a viable, low-cost, and easy-to-use educational tool to support clinical practice, particularly in health education activities, thereby enhancing the self-care competencies and decision-making skills of this population.

## Data Availability

The entire dataset supporting the results of this study was published in the article itself.
