# Supplementary material for: Mobile application for self-care in people with heart failure: a quasi-experimental study
Source: Rev Esc Enferm USP. 2026 Sep 11;60:e20250537. doi: 10.1590/1980-220X-REEUSP-2025-0537en (PMC13566495; doi:10.1590/1980-220X-REEUSP-2025-0537en)
Supplement: Translation

# Translation: Aplicativo móvel no autocuidado de pessoas com insuficiência cardíaca: estudo quase-experimental*

**DOI:** 10.1590/1980-220X-REEUSP-2025-0537pt

**Authors:** Wallison Pereira dos Santos, Mailson Marques de Sousa, André Atanasio Maranhão Almeida, Taciana da Costa Farias Almeida, Fernanda Beatriz Dantas de Freitas, Bernadete de Lourdes André Gouveia, Simone Helena dos Santos Oliveira

**Affiliations:** 1 Universidade Federal de Campina Grande, Centro de Formação de Professores, Unidade Acadêmica de Enfermagem, Cajazeiras, PB, Brasil.; 2 Universidade Federal da Paraíba, Centro de Ciências da Saúde, Departamento de Enfermagem Clínica, João Pessoa, PB, Brasil.; 3 Instituto Federal de Educação, Ciência e Tecnologia da Paraíba, Esperança, PB, Brasil.; 4 Universidade Federal de Campina Grande, Centro de Ciências Biológicas e da Saúde, Unidade Acadêmica de Enfermagem, Campina Grande, PB, Brasil.; 5 Hospital de Emergência e Trauma Senador Humberto Lucena, Departamento de Enfermagem, João Pessoa, PB, Brasil.; 6 Universidade Federal de Campina Grande, Centro de Educação e Saúde, Unidade Acadêmica de Enfermagem, Cuité, PB, Brasil.; 7 Universidade Federal da Paraíba, Centro de Ciências da Saúde, Escola Técnica de Saúde, João Pessoa, PB, Brasil.

**Keywords:** Insuficiência Cardíaca, Autocuidado, Aplicativos Móveis, Tecnologia Biomédica, Enfermagem

## Abstract

**Objetivo:**

Comparar os escores de autocuidado de pessoas com insuficiência cardíaca antes e após o uso do aplicativo móvel “Tum-Tum”.

**Método:**

Estudo quase-experimental, do tipo antes e depois, de grupo único, com amostra de 57 pessoas atendidas em ambulatório de cardiologia de um hospital universitário público na cidade de João Pessoa, Paraíba, realizado entre janeiro e maio de 2024. Para mensurar o autocuidado, utilizou-se o *Self-Care of Heart Failure Index* antes (T0) e depois de 60 dias (T1) de uso do aplicativo móvel. Na análise de dados, foi utilizada estatística descritiva e teste de Wilcoxon para amostras pareadas.

**Resultados:**

54,4% dos participantes eram do sexo masculino. As medianas de idade e anos de estudo foram 60 anos e 6 anos. Verificou-se diferença estatisticamente significativa entre os momentos de pré- (T0) e pós-intervenção (T1) nas subescalas de manutenção e confiança no autocuidado em insuficiência cardíaca.

**Conclusão:**

O uso do aplicativo móvel “Tum-Tum” apresentou influência positiva para o aumento dos escores. O aplicativo apresenta potencial para apoiar estratégias educativas promovidas por profissionais de saúde, sobretudo pelos enfermeiros no autocuidado de pessoas com insuficiência cardíaca. O estudo foi registrado junto ao ReBEC – registro RBR-5zx8rvc.

## INTRODUÇÃO

O autocuidado ocupa um importante papel no controle da insuficiência cardíaca (IC). Como se trata de uma síndrome caracterizada por alterações estruturais e funcionais do coração, que resulta na incapacidade de bombear um volume sanguíneo adequado, é necessária a adesão a uma terapêutica eficaz, que associa tratamento farmacológico e não farmacológico, a fim de atenuar a progressão da síndrome, manter a capacidade funcional e a qualidade de vida relacionada à saúde^(1)^.

O tratamento não farmacológico inclui ações que refletem o autocuidado do indivíduo com IC, como o monitoramento diário do peso corporal, a prática de atividade física regular, a restrição hídrica, a dieta hipossódica, o abandono do tabagismo e do etilismo e a atualização do quadro de imunização^(2)^.

Intervenções educativas são capazes de melhorar os comportamentos de autocuidado a partir da sensibilização e do envolvimento do indivíduo com a sua condição clínica. A educação em saúde liderada por enfermeiros pode resultar em mudanças significativas no estilo de vida do indivíduo. Compreende-se que as intervenções de enfermagem devem estar alicerçadas em evidências científicas sólidas e em um referencial teórico que norteie a prática assistencial, com vistas a promover um cuidado seguro e integral para pessoas com IC^(3,4)^.

A Teoria da Situação Específica do Autocuidado na IC compreende o autocuidado como um processo de tomada de decisão naturalista que envolve a escolha de comportamentos para manter a estabilidade fisiológica (manutenção) e a resposta aos sintomas, quando eles ocorrem. Portanto, o comportamento de autocuidado envolve uma variedade de ações que refletem o engajamento ativo e a responsabilidade pela manutenção, pelo monitoramento e pelo gerenciamento do autocuidado, assim como a confiança para realizá-lo^(5)^. Nessa direção, intervenções baseadas em modelos teóricos para favorecer os comportamentos de autocuidado são oportunas e necessárias.

Na IC, a utilização de recursos tecnológicos tem se mostrado uma ferramenta em expansão. O uso de aplicativos móveis (apps) é empregado como ferramenta adjuvante para intervenções de promoção do autocuidado, monitoramento de sinais e sintomas, controle e manejo terapêutico da doença. Entre os benefícios de seu uso, estão a ausência de restrição de tempo e espaço. No entanto, ainda persistem limitações de acesso à internet e de letramento em saúde (LS) adequado para o uso de tecnologias^(6)^.

O aplicativo “Tum-Tum”, desenvolvido no contexto brasileiro, apresenta evidências adequadas de validade de conteúdo, aparência e usabilidade, com interfaces autoexplicativas que abordam os conceitos sobre a síndrome, as principais causas, os sinais e sintomas, o diagnóstico, o tratamento e as ações de autocuidado para pessoas com IC. A validação de conteúdo foi conduzida por um painel de seis enfermeiros especialistas e resultou em um Índice de Validade de Conteúdo global de 88,4%. A validação de aparência junto ao público-alvo contemplou a organização do aplicativo, as ilustrações, as figuras, o tamanho de fonte, o conteúdo, a linguagem e a motivação para o uso^(6)^. A avaliação de usabilidade obteve escores satisfatórios, com concordância dos participantes quanto à clareza, à linguagem, à facilidade de manuseio e à utilidade, além da indicação do aplicativo para outros usuários^(7)^.

O “Tum-Tum” fornece funcionalidades para o monitoramento de sinais e sintomas de descompensação clínica, registro do peso corporal diário, lembrete das medicações em uso, de exames e de consultas agendadas. A utilização do aplicativo pode ser feita sem a necessidade de rede de internet, o que favorece o uso em qualquer lugar e a qualquer momento^(6,7)^.

É pertinente avaliar possíveis efeitos e desdobramentos do uso de tecnologias em saúde na promoção do autocuidado na população brasileira com IC, levando em consideração altos índices de morbimortalidade, taxa de ocupação de leitos hospitalares, oneração de cofres públicos, assim como impacto na qualidade de vida de pessoas que convivem com a doença^(8)^. Nessa perspectiva, os resultados desta pesquisa poderão favorecer a implantação e implementação de estratégias educativas coadjuvantes no cuidado a esse público. Este estudo teve como objetivo comparar os escores de autocuidado de pessoas com IC mensurados antes e após o uso do aplicativo móvel “Tum-Tum”.

## MÉTODO

### Tipo do Estudo

Trata-se de estudo quase-experimental, de grupo único, do tipo antes e depois, estruturado em observância às recomendações do *Transparent Reporting of Evaluations with Nonrandomized Designs*.

### Local do Estudo

A pesquisa foi realizada no ambulatório de cardiologia de um hospital universitário público do município de João Pessoa, Paraíba, Brasil.

### Critérios de Elegibilidade

Os participantes elegíveis foram aqueles com diagnóstico clínico de IC crônica, em acompanhamento ambulatorial e que apresentaram LS adequado (≥ 19 pontos) pela escala *Eight-Item Health Literacy* ^(9)^. Essa escala avalia a capacidade de obter, processar e interpretar informações básicas em saúde para subsidiar as decisões adequadas. A restrição da elegibilidade a indivíduos com LS adequado visou assegurar o uso autônomo do aplicativo, sem a necessidade de auxílio de terceiros, minimizando as interferências externas capazes de comprometer a fidedignidade das informações registradas.

### População e Amostra

A população do estudo foi composta por pessoas com IC em acompanhamento. A amostra foi calculada por meio do programa de domínio público “Cálculo Amostral”, tendo como base uma margem de erro de 5%, grau de confiabilidade de 95% e desvio padrão estimado na população de 15,7, identificado em estudo prévio com pacientes brasileiros^(10)^, sendo necessário um mínimo de 41 participantes com IC.

Foram incluídos na pesquisa pessoas com IC crônica com idade ≥ 18 anos, que possuíssem *smartphone* com sistema operacional *Android*, estavam em uso de tratamento farmacológico e orientadas no tempo e espaço. A orientação do tempo e espaço foi avaliada pelo pesquisador principal por meio dos seguintes questionamentos: qual é o seu nome? Qual a sua idade? Qual é o dia, mês, ano e local em que estamos? Qual é a cidade onde você mora?^(11)^.

Não foram incluídos os indivíduos que, por motivos de descompensação clínica, passaram por internação prévia nos últimos 30 dias, estavam na fila de transplante cardíaco e/ou indivíduos com planejamento de revascularização do miocárdio ou procedimento intervencionista do tipo *transcatheter aortic valve implantion*, uma vez que são pacientes que apresentam frequentes quadros de instabilidade clínica, o que impossibilitaria a etapa de coleta de dados. Durante o desenvolvimento da pesquisa, houve a viabilidade para alcançar a amostra final de 57 participantes.

### Período e Estratégia de Coleta de Dados

O estudo foi desenvolvido no período de janeiro a maio de 2024. Os indivíduos com IC foram abordados presencialmente na sala de espera, em dia de consulta médica ambulatorial, conforme agendamento prévio. Realizou-se o *download* do aplicativo no *smartphone* do participante, que então teve acesso à sua interface e às suas funcionalidades, como inserir lembretes de medicamentos em uso, consultas e registro de sinais e sintomas. Também houve orientação verbal sobre a importância da adoção de comportamentos de autocuidado para o controle clínico da IC, que também estão presentes no aplicativo. Ressalta-se que o *link* de acesso ao aplicativo foi enviado por meio da ferramenta *WhatsApp*®, deixando o número telefônico registrado para posterior contato.

Em seguida, os participantes realizaram o fluxo de navegação do aplicativo e foram orientados a receber dois contatos telefônicos subsequentes, aos 15 e 30 dias após o *download*, com uma duração aproximada de dez minutos cada, destinados a identificar dúvidas e dificuldades relacionadas exclusivamente à operacionalização da ferramenta. Não foram investigados dados sobre a condição clínica. Os contatos foram conduzidos por roteiro semiestruturado, composto pelos seguintes questionamentos: o(a) senhor(a) apresenta alguma dificuldade de acesso ao aplicativo? Em caso afirmativo, qual? Nos últimos 15/30 dias, quantas vezes utilizou o aplicativo? Quais funcionalidades têm atendido às suas necessidades? Identifica necessidade de melhorias na interface? Possui alguma sugestão?

A intervenção foi o uso do aplicativo móvel “Tum-Tum”, com duração de oito semanas após o momento de recrutamento (T0), finalizada 60 dias após uso do aplicativo (T1). Destaca-se que o período de 60 dias é considerado um intervalo provável para a ocorrência de modificações comportamentais de autocuidado em saúde^(12)^. O “Tum-Tum” é um protótipo de aplicativo para oferecer informações educativas com foco no desenvolvimento do autocuidado na IC por meio da promoção do conhecimento. O aplicativo contém informações sobre a IC, as causas, os sintomas, o diagnóstico e o tratamento, organizado em tópicos educativos que contemplam peso, alimentação, exercícios, atividade sexual, medicamentos, saúde mental, sono, vacinação e hábito de fumar^(6,7)^. As telas do aplicativo estão dispostas na Figura 1.

**Figura 1:** Telas do aplicativo móvel “Tum-Tum” – João Pessoa, PB, Brasil, 2024.

Fonte: Interface “Tum-Tum”, 2024.

Para a coleta de informações sociodemográficas e clínicas, foi utilizado um formulário contendo questões relacionadas às condições de indivíduos com IC, utilizado em estudo prévio^(6)^.

O nível de autocuidado foi mensurado presencialmente no ambulatório por meio da versão brasileira do *Self-Care of Heart Failure Index* (SCHFI)^(13,14)^ nos momentos pré- (T0) e pós-intervenção (T1) com o aplicativo móvel. O SCHFI avalia os comportamentos de autocuidado por meio de 22 itens distribuídos, em três subescalas: manutenção (dez itens); manejo (seis itens); e confiança no autocuidado (seis itens). Cada subescala deve ser analisada de forma independente, sendo escores ≥ 70 pontos indicativos de autocuidado adequado^(13,14)^.

### Análise de Dados

Para as análises estatísticas, os dados foram compilados e, então, processados pelo *software* estatístico R. Utilizou-se estatística descritiva, com apresentação de frequências simples absolutas e percentuais para as variáveis categóricas. Em seguida, foi aplicado o teste de aderência qui-quadrado para verificar a adequabilidade do modelo probabilístico aos dados da pesquisa.

Os dados apresentaram uma distribuição não normal. As variáveis numéricas foram descritas por meio da mediana e do intervalo interquartil (1º quartil – 3º quartil). O diagrama de violino, também conhecido como diagrama de *boxplot*, é apresentado com o objetivo de permitir a visualização dos dados pareados nos momentos T0 e T1, com a localização dos indivíduos nesse intervalo, apresentando os valores médios, o primeiro quartil, a mediana e o terceiro quartil.

A análise do autocuidado por meio da SCHFI se baseou na mensuração da pontuação obtida individualmente em cada subescala (manutenção, manejo e confiança), sendo que valores ≥ 70 pontos são compatíveis com o autocuidado adequado. Com o objetivo de comparar as possíveis diferenças no escore de autocuidado após o uso do aplicativo entre as medianas no momento inicial (T0) e após 60 dias (T1), foi aplicado o teste não paramétrico de Wilcoxon, método de comparação de duas amostras pareadas. Para todas as análises, foi adotado o nível de significância de 5%, com valor de p < 0,05.

### Aspectos Éticos

O estudo foi norteado conforme Resolução nº 466/2012 do Conselho Nacional de Saúde do Ministério da Saúde. Foi aprovado pelo Comitê de Ética em Pesquisa do Hospital Universitário Lauro Wanderley da Universidade Federal da Paraíba (Certificado de Apresentação para Apreciação Ética nº 74304723.1.0000.5183), sob Parecer nº 6.417.699/2023. Além disso, o estudo foi registrado junto à *International Clinical Trials Registration Platform*, por meio do Registro Brasileiro de Ensaios Clínicos (ReBEC – registro RBR-5zx8rvc).

## RESULTADOS

Foram convidados a participar 73 potenciais candidatos, dos quais 13 recusaram. Dos 60 incluídos, três não concluíram o acompanhamento, resultando em uma amostra final de 57 pessoas com IC em acompanhamento no ambulatório de cardiologia. Quanto ao perfil sociodemográfico, as medianas das variáveis idade, anos de estudo e escore de LS foram, respectivamente, 60 anos, 6 anos e 27 pontos. Observou-se um predomínio de participantes do sexo masculino (n = 31; 54,4%), que se autodeclararam brancos (n = 19; 33,3%), residentes em João Pessoa (n = 39; 68,4%), casados ou em união estável (n = 32; 56,1%), que moram com o companheiro (n = 29; 50,9%), aposentados (n = 27; 47,4%) e com renda familiar entre um e dois salários mínimos (n = 45; 78,9%).

A maioria dos participantes possuía fração de ejeção de ventrículo esquerdo reduzida (n = 35; 61,4%), categorizados na classe funcional II da *New York Heart Association* (n = 31; 54,4%) e IC de etiologia isquêmica (n = 32; 56,2%). Em relação às comorbidades associadas à IC, a hipertensão arterial sobressaiu (n = 49; 86,0%), e a classe medicamentosa mais utilizada incluiu os betabloqueadores (atenolol, carvedilol, metoprolol, propranolol e bisoprolol) (n = 43; 75,4%). A Figura 2 apresenta o fluxograma de coleta de dados.

**Figura 2:** Fluxograma de coleta de dados – João Pessoa, PB, Brasil, 2024.

Fonte: Dados da coleta, 2024.

A Tabela 1 apresenta o escore do nível de autocuidado de cada subescala em T0 e T1. Verifica-se que houve aumento nos níveis de cada subescala após o uso do aplicativo “Tum-Tum”. Observou-se uma diferença estatisticamente significativa entre T0 e T1 nos componentes manutenção e confiança do autocuidado.

**Tabela 1:** Escores dos componentes do *Self-Care of Heart Failure Index* em T0 (pré-intervenção) e T1 (pós-intervenção) – João Pessoa, PB, Brasil, 2024.

Subescalas	T0 – Pré-intervenção			T1 – Pós-intervenção		Valor de p*
	Mediana	(Q1–Q3)		Mediana	(Q1–Q3)	
Manutenção	63	(46–76)		66	(63–72)	0,002
Manejo	59	(49–70)		62	(54–64)	0,063
Confiança	85	(72–81)		95	(72–95)	0,001

Notas: *Teste pareado de Wilcoxon.

Fonte: Dados da pesquisa (2024).

Ao comparar os valores da subescala de manutenção do autocuidado na Figura 3, expressos no boxplot, foi possível verificar os dados pareados e a localização dos indivíduos no primeiro momento (T0), com mediana de 63, e no segundo momento (T1), com mediana de 66, após o uso do aplicativo móvel, com uma mudança estatisticamente significativa. Além disso, houve mudança na dispersão dos participantes, que se concentraram mais em valores superiores a 60 pontos após o uso do “Tum-Tum”.

**Figura 3:** Comparação dos escores das subescalas de manutenção, manejo e confiança do autocuidado entre T0 (pré-intervenção) e T1 (pós-intervenção) – João Pessoa, PB, Brasil, 2024.

Fonte: Dados da pesquisa (2024).

Na subescala de manejo do autocuidado, é possível observar que, antes do uso do aplicativo móvel, os valores estavam dispersos, apresentando escores menores que 40 pontos (T0). Entretanto, após a intervenção em T1, os valores se concentraram acima de 50 pontos. Apesar de não ter sido identificada significância estatística, observa-se que houve aumento nos valores da subescala de manejo.

A comparação entre os valores pareados da subescala de confiança no autocuidado no momento de pré-intervenção (T0) e no momento de pós-intervenção (T1) revela que, no momento de pré-intervenção, os valores são adequados, com escores ≥ 70 pontos. Após a intervenção, os valores se concentram em ≥ 90 pontos, o que evidencia o aumento da confiança para o autocuidado dos participantes após o uso do aplicativo móvel, com respostas agrupadas na margem superior, ou seja, em escores elevados, entre 90 e 100 pontos.

A comparação entre os escores de cada subescala do SCHIF, entre os momentos T0 e T1, está disposta na Figura 3.

## DISCUSSÃO

Neste estudo, observou-se que, no primeiro momento (T0), os escores de manutenção e manejo do autocuidado se apresentaram insatisfatórios, e no momento pós-intervenção (T1), o nível de autocuidado progrediu, sobretudo na subescala de manutenção, a qual reflete os comportamentos que objetivam a estabilidade fisiológica.

O autocuidado é resultante da integração entre os comportamentos de manutenção, manejo e confiança, ou seja, é necessário que o indivíduo detenha domínio desses componentes para alcançar um autocuidado adequado, com potencial para prevenir e mitigar complicações oriundas de quadros de descompensação clínica, o que, por sua vez, predispõe o indivíduo a reinternações frequentes e desfechos adversos em saúde^(5)^.

A manutenção do autocuidado constitui uma prática proativa, na qual o indivíduo adota comportamentos voltados à prevenção de complicações e à preservação da estabilidade fisiológica, englobando hábitos saudáveis e adesão ao tratamento medicamentoso^(5)^. Observou-se que, após o uso do aplicativo móvel “Tum-Tum”, os participantes apresentaram um aumento relevante e estatisticamente significativo na subescala de manutenção, sugerindo que, após o uso do aplicativo, adotaram comportamentos com o objetivo de manter a estabilidade fisiológica. Entretanto, ainda permaneceram abaixo do ponto de corte considerado satisfatório (≥ 70 pontos).

O manejo do autocuidado envolve uma resposta reativa, na qual o indivíduo reconhece sinais e sintomas e toma decisões frente a eles. Nesse contexto, o manejo dos sintomas representa a interface entre a identificação de alterações clínicas e a tomada de decisão adequada, refletindo uma atitude proativa em que o paciente seleciona o comportamento de autocuidado mais apropriado à sua condição^(5)^.

Em pacientes com IC, a percepção dos sintomas é uma tarefa difícil devido à influência de múltiplas comorbidades que podem afetar as sensações corporais de outras condições não relacionadas à IC^(13)^. É possível inferir que as pessoas identificam os sinais e sintomas característicos, porém a tomada de decisão se restringe ao uso de medicamentos orais para o controle dos sintomas da doença, ou seja, o componente manejo do autocuidado não é exercido em sua plenitude.

Uma investigação conduzida em São Paulo corrobora a afirmação anterior, uma vez que todos os participantes apresentaram escores inadequados nas três subescalas, em graus variados, com destaque para o manejo do autocuidado como a de menor pontuação^(10)^. Adicionalmente, estudo realizado na Suíça identificou um padrão semelhante, com o manejo do autocuidado correspondendo à subescala de menor pontuação e a confiança atingindo escores adequados^(15)^. Em contraposição, investigação conduzida em Portugal identificou a subescala de confiança como a de menor pontuação^(16)^.

A Teoria da Situação Específica do Autocuidado em IC considera que os seres humanos almejam ser saudáveis, tanto fisicamente quanto mentalmente, e, por isso, depositam sua confiança em determinadas condições terapêuticas^(5)^. No momento basal (T0), a subescala de confiança no autocuidado já se mostrou satisfatória e, após a intervenção (T1), observou-se uma elevação do escore, com a manutenção de valores adequados. Evidencia-se que a maior confiança no autocuidado se associa a melhores comportamentos de autocuidado^(5)^. Esse achado é consistente com os resultados do presente estudo, nos quais o aumento do escore de confiança acompanhou a elevação dos escores de manutenção e manejo.

É importante refletir que a confiança no autocuidado influencia diretamente a manutenção e o manejo do autocuidado, uma vez que o indivíduo manifesta e deposita sua confiança unicamente no tratamento medicamentoso, não se atentando à necessidade de modificar hábitos e comportamentos que o façam alcançar o comportamento de autocuidado de maneira satisfatória.

Estudos prévios apresentam achados semelhantes, evidenciando um autocuidado insatisfatório em pessoas com IC^(10,16,17)^. Revisão sistemática com meta-análise apontou um autocuidado inadequado em todas as três dimensões do autocuidado: manutenção, manejo e confiança^(18)^. No cenário brasileiro, os dados encontrados no estudo em questão, no momento pré-intervenção, nas subescalas de manutenção e manejo do autocuidado, foram inferiores a 70 pontos, sendo caracterizados como inadequados. Entretanto, a subescala de confiança obteve um escore superior a 70, sendo considerada adequada^(19)^.

Compreender que os componentes do autocuidado são influenciadores e influenciados por fatores externos e internos faz com que o cuidado com a pessoa que convive com a IC seja holístico, complexo, integral e, ao mesmo tempo, individualizado, levando em consideração o contexto social, econômico e cultural no qual o indivíduo se insere^(20)^. Os comportamentos de manutenção e manejo do autocuidado são influenciados diretamente pelas variáveis conhecimento, LS e apoio social^(17,18)^.

O autocuidado envolve responsabilidade individual, autonomia e independência, incluindo a capacidade de se engajar em sua prática de cuidado consigo mesmo^(18)^. É possível aumentar os níveis de autocuidado e conhecimento a partir de estratégias educativas com o uso de tecnologias da informação. Ensaio clínico randomizado, que realizou um treinamento intensivo de quatro dias e ciclos a cada quatro meses, com duração de 12 meses, por meio de folhetos educativos e ilustrados, observou um aumento nos escores de autocuidado do grupo intervenção quando comparado ao grupo controle^(21)^.

Nessa perspectiva, ressalta-se a importância de intervenções educativas, como a desenvolvida neste estudo, para a promoção do autocuidado. Investigação realizada no Japão, que examinou o efeito de um aplicativo móvel no comportamento de autocuidado de pacientes com C crônica após dois meses de uso, por meio da *European Heart Failure Self-Care Behaviour Scale*, corroborou os resultados desta pesquisa ao evidenciar melhora no autocuidado do grupo de intervenção^(22)^.

Na China, estudo quase-experimental observou que o uso de aplicativo móvel melhorou a compreensão e a conscientização dos pacientes sobre a IC diante das habilidades de autogestão da síndrome^(23)^. Na Coreia do Sul, estudo piloto, cuja intervenção foi baseada em aplicativo móvel, resultou em melhora significativa dos escores de LS digital, do conhecimento sobre a doença e dos comportamentos de autocuidado em pacientes com IC^(24)^. Outra investigação evidenciou uma melhora significativa na qualidade do autocuidado após três meses de uso do aplicativo móvel^(25)^.

Meta-análise com o objetivo de avaliar o impacto das intervenções de e-saúde no manejo da doença em pacientes com IC, incluindo ensaios clínicos controlados e randomizados de diversos países (China, EUA, Reino Unido, Suécia, Austrália), sugere que as intervenções em saúde pautadas na tecnologia da informação são replicáveis em diversas outras realidades, tendo como desfecho principal a melhora no prognóstico de indivíduos com a doença e capazes de reduzir a mortalidade, reinternações e favorecer a melhoria na qualidade de vida^(26)^.

No presente estudo, os participantes apresentaram melhora nos escores de manutenção, manejo e confiança no autocuidado. O referencial teórico adotado postula que os comportamentos de manutenção são influenciados por fatores externos e que a tomada de decisão pode ocorrer de forma consciente ou inconsciente, a partir da interação entre características individuais e o contexto em que o indivíduo está inserido^(5)^. Infere-se, portanto, que a exposição ao aplicativo exerceu influência positiva no desempenho do autocuidado, com potencial para ser incorporado como ferramenta de apoio nas estratégias educativas conduzidas por enfermeiros e pela equipe multidisciplinar.

Entre as limitações do presente estudo, destacam-se o perfil restrito dos participantes com níveis adequados de LS e a natureza multifatorial do autocuidado, influenciado por condições impostas pela própria síndrome, idade avançada, baixa escolaridade, aspectos psicológicos e apoio social insuficiente^(1,5)^. Tais características limitam a generalização dos achados, que devem ser interpretados com cautela. Adicionalmente, o autorrelato do uso do aplicativo está sujeito ao viés de desejabilidade social e à ausência de métricas objetivas de engajamento, acesso e tempo de uso, o que constitui uma limitação adicional a ser superada em investigações futuras. Recomenda-se a realização de ensaios clínicos randomizados com amostras mais amplas, por um período mais longo e em diferentes contextos assistenciais, utilizando o aplicativo “Tum-Tum” para ampliar a comparabilidade dos resultados e avaliar a eficácia da ferramenta.

## CONCLUSÃO

Este estudo identificou um aumento nos escores de manutenção, manejo e confiança no autocuidado de pessoas com insuficiência cardíaca após o uso do aplicativo móvel “Tum-Tum”. Os resultados fornecem aos profissionais de saúde, sobretudo aos enfermeiros, uma ferramenta educativa viável, de baixo custo e de fácil manuseio para apoiar a prática assistencial, especialmente as atividades de educação em saúde, potencializando as competências e as habilidades para o autocuidado e a tomada de decisão nessa população.

## Data Availability

Todo o conjunto de dados que dá suporte aos resultados deste estudo foi publicado no próprio artigo.

# Translation: Aplicación móvil para el autocuidado de personas con insuficiencia cardíaca: estudio cuasi-experimental*

**DOI:** 10.1590/1980-220X-REEUSP-2025-0537es

**Authors:** Wallison Pereira dos Santos, Mailson Marques de Sousa, André Atanasio Maranhão Almeida, Taciana da Costa Farias Almeida, Fernanda Beatriz Dantas de Freitas, Bernadete de Lourdes André Gouveia, Simone Helena dos Santos Oliveira

**Affiliations:** 1 Universidade Federal de Campina Grande, Centro de Formação de Professores, Unidade Acadêmica de Enfermagem, Cajazeiras, PB, Brasil.; 2 Universidade Federal da Paraíba, Centro de Ciências da Saúde, Departamento de Enfermagem Clínica, João Pessoa, PB, Brasil.; 3 Instituto Federal de Educação, Ciência e Tecnologia da Paraíba, Esperança, PB, Brasil.; 4 Universidade Federal de Campina Grande, Centro de Ciências Biológicas e da Saúde, Unidade Acadêmica de Enfermagem, Campina Grande, PB, Brasil.; 5 Hospital de Emergência e Trauma Senador Humberto Lucena, Departamento de Enfermagem, João Pessoa, PB, Brasil.; 6 Universidade Federal de Campina Grande, Centro de Educação e Saúde, Unidade Acadêmica de Enfermagem, Cuité, PB, Brasil.; 7 Universidade Federal da Paraíba, Centro de Ciências da Saúde, Escola Técnica de Saúde, João Pessoa, PB, Brasil.

**Keywords:** Insuficiencia Cardíaca, Autocuidado, Aplicaciones Móviles, Tecnología Biomédica, Enfermería

## Abstract

**Objetivo:**

Comparar las puntuaciones de autocuidado de personas con insuficiencia cardíaca antes y después de usar la aplicación móvil “Tum-Tum”.

**Método:**

Estudio cuasiexperimental, pre-post, de un solo grupo con una muestra de 57 personas atendidas en una clínica ambulatoria de cardiología de un hospital universitario público en la ciudad de João Pessoa, Paraíba, realizado entre enero y mayo de 2024. Para medir el autocuidado, se utilizó el Self-Care of Heart Failure Index antes (T0) y después de 60 días (T1) de usar la aplicación móvil. En el análisis de datos se utilizaron estadísticas descriptivas y la prueba de Wilcoxon para muestras pareadas.

**Resultados:**

El 54,4% de los participantes eran hombres. La mediana de edad y de años de educación fue de 60 y 6 años, respectivamente. Se encontró una diferencia estadísticamente significativa entre los momentos previos (T0) y posteriores a la intervención (T1) en las subescalas de mantenimiento y confianza para el autocuidado en la insuficiencia cardíaca.

**Conclusión:**

El uso de la aplicación móvil “Tum-Tum” demostró una influencia positiva en el aumento de las puntuaciones. La aplicación tiene el potencial de apoyar las estrategias educativas promovidas por los profesionales de la salud, especialmente el personal de enfermería, en el autocuidado de las personas con insuficiencia cardíaca. El estudio se registró en ReBEC con el número RBR-5zx8rvc.

## INTRODUCCIÓN

El autocuidado desempeña un papel importante en el control de la insuficiencia cardíaca (IC). Dado que se trata de un síndrome caracterizado por alteraciones estructurales y funcionales del corazón, que da lugar a la incapacidad de bombear un volumen sanguíneo adecuado, es necesario seguir un tratamiento eficaz, que combine medidas farmacológicas y no farmacológicas, con el fin de mitigar la progresión del síndrome, mantener la capacidad funcional y la calidad de vida relacionada con la salud^(1)^.

El tratamiento no farmacológico incluye medidas que reflejan el autocuidado de la persona con IC, como el control diario del peso corporal, la práctica de actividad física regular, la restricción de líquidos, la dieta baja en sodio, dejar de fumar y de consumir alcohol, y poner al día el calendario de vacunación^(2)^.

Las intervenciones educativas pueden mejorar los comportamientos de autocuidado a partir de la sensibilización y la implicación del paciente con su condición clínica.

La educación en salud dirigida por enfermeros puede dar lugar a cambios significativos en el estilo de vida de la persona. Se entiende que las intervenciones de enfermería deben basarse en evidencia científica sólida y en un marco teórico que oriente la práctica asistencial, con el fin de promover una atención segura e integral para las personas con IC^(3,4)^.

La Teoría de la Situación Específica del Autocuidado en la IC concibe el autocuidado como un proceso de toma de decisiones naturalista que implica la elección de conductas para mantener la estabilidad fisiológica (mantenimiento) y la respuesta a los síntomas, cuando estos se presentan. Por lo tanto, el comportamiento de autocuidado implica una variedad de acciones que reflejan el compromiso activo y la responsabilidad por el mantenimiento, el monitoreo y el manejo del autocuidado, así como la confianza para llevarlo a cabo^(5)^. En este sentido, las intervenciones basadas en modelos teóricos para fomentar los comportamientos de autocuidado son oportunas y necesarias.

En la IC, el uso de recursos tecnológicos se ha convertido en una herramienta en expansión. Las aplicaciones móviles (apps) se emplean como herramienta complementaria para intervenciones de promoción del autocuidado, monitoreo de signos y síntomas, control y manejo terapéutico de la enfermedad. Entre los beneficios de su uso se encuentra la ausencia de restricciones de tiempo y espacio. Sin embargo, aún persisten limitaciones en el acceso a Internet y en la alfabetización en salud (AS) adecuada para el uso de las tecnologías^(6)^.

La aplicación «Tum-Tum», desarrollada en el contexto brasileño, presenta evidencia adecuada de validez de contenido, apariencia y usabilidad, con interfaces intuitivas que abordan los conceptos sobre el síndrome, las principales causas, los signos y síntomas, el diagnóstico, el tratamiento y las medidas de autocuidado para personas con IC. La validación del contenido fue realizada por un panel de seis enfermeros especialistas y arrojó un Índice de Validez del Contenido global del 88,4%. La validación de la apariencia con el público objetivo abarcó la organización de la aplicación, las ilustraciones, las figuras, el tamaño de la fuente, el contenido, el lenguaje y la motivación para su uso^(6)^. La evaluación de usabilidad obtuvo calificaciones satisfactorias, con coincidencia entre los participantes en cuanto a la claridad, el lenguaje, la facilidad de uso y la utilidad, además de la recomendación de la aplicación a otros usuarios^(7)^.

La aplicación «Tum-Tum» ofrece funciones para el monitoreo de signos y síntomas de descompensación clínica, el registro diario del peso corporal, recordatorios de los medicamentos que se están tomando, de los exámenes y de las consultas programadas. La aplicación se puede utilizar sin necesidad de conexión a Internet, lo que facilita su uso en cualquier lugar y en cualquier momento^(6,7)^.

Es pertinente evaluar los posibles efectos y consecuencias del uso de tecnologías en salud en la promoción del autocuidado entre la población brasileña con IC, tomando en cuenta los altos índices de morbilidad y mortalidad, la tasa de ocupación de camas hospitalarias, la carga sobre las arcas públicas, así como el impacto en la calidad de vida de las personas que conviven con la enfermedad^(8)^.

Desde esta perspectiva, los resultados de esta investigación podrían favorecer la adopción e implementación de estrategias educativas complementarias en la atención a este público. El objetivo de este estudio fue comparar los puntajes de autocuidado de las personas con IC medidos antes y después del uso de la aplicación móvil «Tum-Tum».

## MÉTODO

### Tipo de Estudio

Se trata de un estudio cuasi-experimental, de grupo único, de tipo antes y después, estructurado de acuerdo con las recomendaciones del *Transparent Reporting of Evaluations with Nonrandomized Designs*.

### Lugar del Estudio

La investigación se llevó a cabo en la consulta de cardiología de un hospital universitario público del municipio de João Pessoa, Paraíba, Brasil.

### Criterios de Elegibilidad

Los participantes elegibles fueron aquellos con diagnóstico clínico de IC crónica, en seguimiento ambulatorio y que presentaron un nivel adecuado de alfabetización en salud (≥ 19 puntos) según la escala *Eight-Item Health Literacy* ^(9)^. Esta escala evalúa la capacidad de obtener, procesar e interpretar información básica sobre salud para tomar decisiones adecuadas. La restricción de la elegibilidad a individuos con un nivel adecuado de AS tuvo como objetivo garantizar el uso autónomo de la aplicación, sin necesidad de ayuda de terceros, minimizando así las interferencias externas que pudieran comprometer la fiabilidad de la información registrada.

### Población y Muestra

La población del estudio estuvo compuesta por personas con insuficiencia cardíaca en seguimiento. La muestra se calculó mediante el programa de dominio público «Cálculo Amostral», basándose en un margen de error del 5%, un nivel de confianza del 95% y una desviación estándar estimada en la población de 15,7, identificada en un estudio previo con pacientes brasileños^(10)^, lo que requirió un mínimo de 41 participantes con IC.

Se incluyeron en la investigación personas con IC crónica de 18 años o más, que tuvieran un *smartphone* con sistema operativo *Android*, estuvieran en tratamiento farmacológico y tuvieran orientación temporal y espacial. La orientación temporal y espacial fue evaluada por el investigador principal mediante las siguientes preguntas: ¿Cómo te llamas? ¿Qué edad tienes? ¿Qué día, mes, año y lugar es este? ¿En qué ciudad vive?^(11)^.

No se incluyeron los individuos que, por motivos de descompensación clínica, habían sido hospitalizados previamente en los últimos 30 días, se encontraban en lista de espera para un trasplante cardíaco y/o aquellos con una programación de revascularización miocárdica o un procedimiento intervencionista del tipo *implante transcatéter de válvula aórtica*, ya que se trata de pacientes que presentan cuadros frecuentes de inestabilidad clínica, lo que imposibilitaría la etapa de recolección de datos. Durante el desarrollo de la investigación, se logró alcanzar la muestra final de 57 participantes.

### Período y Estrategia de Recolección de Datos

El estudio se llevó a cabo entre enero y mayo de 2024. Se abordó a las personas con insuficiencia cardíaca (IC) en persona en la sala de espera, el día de su consulta médica ambulatoria, según cita previa. Se realizó la *descarga* de la aplicación en el *smartphone* del participante, quien luego tuvo acceso a su interfaz y a sus funcionalidades, como ingresar recordatorios de los medicamentos que toma, sus citas y el registro de signos y síntomas. También se brindó orientación verbal sobre la importancia de adoptar comportamientos de autocuidado para el control clínico de la IC, los cuales también están presentes en la aplicación. Cabe destacar que el *enlace* de acceso a la aplicación se envió a través de la herramienta *WhatsApp*®, dejando registrado el número de teléfono para contacto posterior.

A continuación, los participantes recorrieron el flujo de navegación de la aplicación y se les informó que recibirían dos llamadas telefónicas posteriores, a los 15 y 30 días después de la *descarga*, con una duración aproximada de diez minutos cada una, destinadas a identificar dudas y dificultades relacionadas exclusivamente con el uso de la herramienta. No se recopilaron datos sobre la condición clínica. Los contactos se llevaron a cabo siguiendo un guion semiestructurado, compuesto por las siguientes preguntas: *¿Tiene alguna dificultad para acceder a la aplicación? En caso afirmativo, ¿cuál? En los últimos 15/30 días, ¿cuántas veces utilizó la aplicación? ¿Qué funcionalidades han satisfecho sus necesidades? ¿Identifica la necesidad de mejoras en la interfaz? ¿Tiene alguna sugerencia?*

La intervención consistió en el uso de la aplicación móvil «Tum-Tum», con una duración de ocho semanas a partir del momento del reclutamiento (T0), y concluyó 60 días después del inicio del uso de la aplicación (T1). Cabe destacar que el período de 60 días se considera un intervalo probable para que se produzcan cambios conductuales en el autocuidado en salud^(12)^. «Tum-Tum» es un prototipo de aplicación que ofrece información educativa enfocada en el desarrollo del autocuidado en la IC mediante la promoción del conocimiento. La aplicación contiene información sobre la IC, sus causas, síntomas, diagnóstico y tratamiento, organizada en temas educativos que abarcan el peso, la alimentación, el ejercicio, la actividad sexual, los medicamentos, la salud mental, el sueño, la vacunación y el hábito de fumar^(6,7)^. Las pantallas de la aplicación se muestran en la Figura 1.

**Figura 1:** Pantallas de la aplicación móvil «Tum-Tum» – João Pessoa, PB, Brasil, 2024.

Fuente: Interfaz de «Tum-Tum», 2024.

Para la recolección de información sociodemográfica y clínica, se utilizó un formulario con preguntas relacionadas con las condiciones de las personas con insuficiencia cardíaca, el cual ya se había empleado en un estudio previo^(6)^.

El nivel de autocuidado se midió en persona en el consultorio mediante la versión brasileña del *Self-Care of Heart Failure Index* (SCHFI)^(13,14)^ antes (T0) y después (T1) de la intervención con la aplicación móvil. El SCHFI evalúa los comportamientos de autocuidado mediante 22 ítems distribuidos en tres subescalas: mantenimiento (diez ítems); manejo (seis ítems); y confianza en el autocuidado (seis ítems). Cada subescala debe analizarse de manera independiente, y las puntuaciones ≥ 70 puntos indican un autocuidado adecuado^(13,14)^.

### Análisis de Datos

Para los análisis estadísticos, los datos se recopilaron y luego se procesaron mediante el *software* estadístico R. Se utilizó estadística descriptiva, con presentación de frecuencias absolutas simples y porcentuales para las variables categóricas. A continuación, se aplicó la prueba de ajuste Chi-cuadrado para verificar la adecuación del modelo probabilístico a los datos de la investigación.

Los datos presentaron una distribución no normal. Las variables numéricas se describieron mediante la mediana y el intervalo intercuartílico (1.º cuartil – 3.º cuartil). El diagrama de violín, también conocido como diagrama de *boxplot*, se presenta con el objetivo de permitir la visualización de los datos emparejados en los momentos T0 y T1, con la ubicación de los individuos en ese intervalo, mostrando los valores medios, el primer cuartil, la mediana y el tercer cuartil.

El análisis del autocuidado mediante el SCHFI se basó en la medición de la puntuación obtenida individualmente en cada subescala (mantenimiento, manejo y confianza), siendo que los valores ≥ 70 puntos son compatibles con un autocuidado adecuado. Con el objetivo de comparar las posibles diferencias en la puntuación de autocuidado tras el uso de la aplicación entre las medianas en el momento inicial (T0) y después de 60 días (T1), se aplicó la prueba no paramétrica de Wilcoxon, un método de comparación de dos muestras emparejadas. Para todos los análisis, se adoptó un nivel de significación del 5%, con un valor de p < 0,05.

### Aspectos Éticos

El estudio se llevó a cabo de conformidad con la Resolución n.º 466/2012 del Consejo Nacional de Salud del Ministerio de Salud. Fue aprobado por el Comité de Ética en Investigación del Hospital Universitario Lauro Wanderley de la Universidad Federal de Paraíba (Certificado de Presentación para Evaluación Ética n.º 74304723.1.0000.5183), mediante el Dictamen n.º 6.417.699/2023.

Además, el estudio se registró en la *International Clinical Trials Registration Platform*, a través del Registro Brasileño de Ensayos Clínicos (ReBEC – registro RBR-5zx8rvc).

## RESULTADOS

Se invitó a participar a 73 candidatos potenciales, de los cuales 13 se negaron. De los 60 incluidos, tres no completaron el seguimiento, lo que dio como resultado una muestra final de 57 personas con IC en seguimiento en el consultorio de cardiología. En cuanto al perfil sociodemográfico, las medianas de las variables edad, años de estudio y puntaje de AS fueron, respectivamente, 60 años, 6 años y 27 puntos. Se observó un predominio de participantes del sexo masculino (n = 31; 54,4%), quienes se autodefinieron como blancos (n = 19; 33,3%), residentes en João Pessoa (n = 39; 68,4%), casados o en unión estable (n = 32; 56,1%), que vivían con su pareja (n = 29; 50,9%), jubilados (n = 27; 47,4%) y con un ingreso familiar de entre uno y dos salarios mínimos (n = 45; 78,9%).

La mayoría de los participantes presentaba una fracción de eyección del ventrículo izquierdo reducida (n = 35; 61,4%), estaban clasificados en la clase funcional II de la *New York Heart Association* (n = 31; 54,4%) y presentaban IC de etiología isquémica (n = 32; 56,2%). En cuanto a las comorbilidades asociadas a la IC, destacó la hipertensión arterial (n = 49; 86,0%), y la clase de medicamentos más utilizada incluyó los betabloqueadores (atenolol, carvedilol, metoprolol, propranolol y bisoprolol) (n = 43; 75,4%). La Figura 2 presenta el diagrama de flujo de la recolección de datos.

**Figura 2:** Diagrama de flujo de la recolección de datos – João Pessoa, PB, Brasil, 2024.

Fuente: Datos de la recolección, 2024.

La Tabla 1 presenta la puntuación del nivel de autocuidado de cada subescala en T0 y T1. Se observa que hubo un aumento en los niveles de cada subescala tras el uso de la aplicación «Tum-Tum». Se observó una diferencia estadísticamente significativa entre T0 y T1 en los componentes de mantenimiento y confianza del autocuidado.

**Tabla 1:** Puntuaciones de los componentes del *Índice de autocuidado de la insuficiencia cardíaca* en T0 (preintervención) y T1 (posintervención) – João Pessoa, PB, Brasil, 2024.

Subescalas	T0 – Pre-intervención			T1 – Post-intervención		Valor de p*
	Mediana	(Q1–Q3)		Mediana	(Q1–Q3)	
Mantenimiento	63	(46–76)		66	(63–72)	0,002
Manejo	59	(49–70)		62	(54–64)	0,063
Confianza	85	(72–81)		95	(72–95)	0,001

Notas: *Test pareado de Wilcoxon.

Fonte: Datos de la investigación (2024).

Al comparar los valores de la subescala de mantenimiento del autocuidado en la Figura 3, expresados en el diagrama de caja, se pudieron verificar los datos emparejados y la ubicación de los participantes en el primer momento (T0), con una mediana de 63, y en el segundo momento (T1), con una mediana de 66, tras el uso de la aplicación móvil, lo que supuso un cambio estadísticamente significativo. Además, se observó un cambio en la dispersión de los participantes, quienes se concentraron más en valores superiores a 60 puntos tras el uso de «Tum-Tum».

**Figura 3:** Comparación de los puntajes de las subescalas de mantenimiento, manejo y confianza en el autocuidado entre T0 (previa a la intervención) y T1 (posterior a la intervención) – João Pessoa, PB, Brasil, 2024.

Fuente: Datos de la investigación (2024).

En la subescala de manejo del autocuidado, se puede observar que, antes del uso de la aplicación móvil, los valores estaban dispersos, presentando puntuaciones inferiores a 40 puntos (T0). Sin embargo, tras la intervención en T1, los valores se concentraron por encima de los 50 puntos. Aunque no se identificó significación estadística, se observa que hubo un aumento en los valores de la subescala de manejo.

La comparación entre los valores emparejados de la subescala de confianza en el autocuidado en el momento previo a la intervención (T0) y en el momento posterior a la intervención (T1) revela que, en el momento previo a la intervención, los valores son adecuados, con puntuaciones ≥ 70 puntos. Tras la intervención, los valores se concentran en ≥ 90 puntos, lo que evidencia el aumento de la confianza en el autocuidado de los participantes tras el uso de la aplicación móvil, con respuestas agrupadas en el rango superior, es decir, en puntuaciones elevadas, entre 90 y 100 puntos.

La comparación entre las puntuaciones de cada subescala del SCHIF, entre los momentos T0 y T1, se presenta en la Figura 3.

## DISCUSIÓN

En este estudio, se observó que, en el primer momento (T0), las puntuaciones de mantenimiento y manejo del autocuidado resultaron insatisfactorias, y en el momento posterior a la intervención (T1), el nivel de autocuidado mejoró, sobre todo en la subescala de mantenimiento, la cual refleja los comportamientos que tienen como objetivo la estabilidad fisiológica.

El autocuidado es el resultado de la integración entre los comportamientos de mantenimiento, manejo y confianza, es decir, es necesario que la persona domine estos componentes para alcanzar un autocuidado adecuado, con el potencial de prevenir y mitigar complicaciones derivadas de cuadros de descompensación clínica, lo que, a su vez, predispone a la persona a reingresos frecuentes y resultados adversos en salud^(5)^.

El mantenimiento del autocuidado constituye una práctica proactiva, en la que la persona adopta comportamientos orientados a la prevención de complicaciones y a la preservación de la estabilidad fisiológica, lo que incluye hábitos saludables y la adherencia al tratamiento farmacológico^(5)^. Se observó que, tras el uso de la aplicación móvil «Tum-Tum», los participantes presentaron un aumento relevante y estadísticamente significativo en la subescala de mantenimiento, lo que sugiere que, tras el uso de la aplicación, adoptaron comportamientos con el objetivo de mantener la estabilidad fisiológica. Sin embargo, aún se mantuvieron por debajo del punto de corte considerado satisfactorio (≥ 70 puntos).

El manejo del autocuidado implica una respuesta reactiva, en la que el individuo reconoce los signos y síntomas y toma decisiones al respecto. En este contexto, el manejo de los síntomas representa la interfaz entre la identificación de alteraciones clínicas y la toma de decisiones adecuadas, lo que refleja una actitud proactiva en la que el paciente selecciona el comportamiento de autocuidado más apropiado para su condición^(5)^.

En pacientes con IC, la percepción de los síntomas es una tarea difícil debido a la influencia de múltiples comorbilidades que pueden afectar las sensaciones corporales de otras afecciones no relacionadas con la IC^(13)^. Es posible deducir que las personas identifican los signos y síntomas característicos; sin embargo, la toma de decisiones se limita al uso de medicamentos orales para el control de los síntomas de la enfermedad; es decir, el componente de autocuidado no se ejerce en su totalidad.

Una investigación realizada en São Paulo corrobora la afirmación anterior, ya que todos los participantes presentaron puntuaciones inadecuadas en las tres subescalas, en distintos grados, destacando que el manejo del autocuidado fue la que obtuvo la puntuación más baja^(10)^.

Además, un estudio realizado en Suiza identificó un patrón similar, en el que el manejo del autocuidado correspondía a la subescala con la puntuación más baja y la confianza alcanzaba puntuaciones adecuadas^(15)^. Por el contrario, una investigación llevada a cabo en Portugal identificó la subescala de confianza como la de menor puntuación^(16)^.

La Teoría de la Situación Específica del Autocuidado en la IC sostiene que los seres humanos aspiran a estar sanos, tanto física como mentalmente, y, por ello, depositan su confianza en determinadas condiciones terapéuticas^(5)^. En el momento inicial (T0), la subescala de confianza en el autocuidado ya se mostró satisfactoria y, tras la intervención (T1), se observó un aumento en la puntuación, manteniendo valores adecuados. Se evidencia que una mayor confianza en el autocuidado se asocia con mejores comportamientos de autocuidado^(5)^. Este hallazgo es consistente con los resultados del presente estudio, en los que el aumento de la puntuación de confianza fue acompañado por el aumento de las puntuaciones de mantenimiento y manejo.

Es importante considerar que la confianza en el autocuidado influye directamente en el mantenimiento y el manejo del autocuidado, ya que la persona manifiesta y deposita su confianza únicamente en el tratamiento farmacológico, sin prestar atención a la necesidad de modificar hábitos y comportamientos que le permitan alcanzar un autocuidado satisfactorio.

Estudios previos presentan hallazgos similares, lo que evidencia un autocuidado insatisfactorio en personas con IC^(10,16,17)^. Una revisión sistemática con metaanálisis señaló un autocuidado inadecuado en las tres dimensiones del autocuidado: mantenimiento, manejo y confianza^(18)^. En el contexto brasileño, los datos obtenidos en el estudio en cuestión, en el momento previo a la intervención, en las subescalas de mantenimiento y manejo del autocuidado, fueron inferiores a 70 puntos, lo que se caracterizó como inadecuado. Sin embargo, la subescala de confianza obtuvo una puntuación superior a 70, por lo que se consideró adecuada^(19)^.

Comprender que los componentes del autocuidado influyen e son influenciados por factores externos e internos hace que el cuidado de la persona que vive con IC sea holístico, complejo, integral y, al mismo tiempo, individualizado, tomando en cuenta el contexto social, económico y cultural en el que se inserta el individuo^(20)^. Los comportamientos de mantenimiento y manejo del autocuidado se ven influenciados directamente por las variables de conocimiento, AS y apoyo social^(17,18)^.

El autocuidado implica responsabilidad individual, autonomía e independencia, incluyendo la capacidad de comprometerse con la práctica del cuidado de sí mismo^(18)^. Es posible aumentar los niveles de autocuidado y conocimiento mediante estrategias educativas que utilicen tecnologías de la información. Un ensayo clínico aleatorizado, que llevó a cabo una capacitación intensiva de cuatro días y ciclos cada cuatro meses, con una duración de 12 meses, mediante folletos educativos e ilustrados, observó un aumento en las puntuaciones de autocuidado del grupo de intervención en comparación con el grupo de control^(21)^.

Desde esta perspectiva, se destaca la importancia de las intervenciones educativas, como la desarrollada en este estudio, para promover el autocuidado. Una investigación realizada en Japón, que examinó el efecto de una aplicación móvil en el comportamiento de autocuidado de pacientes con insuficiencia cardíaca crónica tras dos meses de uso, mediante la *European Heart Failure Self-Care Behaviour Scale*, corroboró los resultados de esta investigación al evidenciar una mejora en el autocuidado del grupo de intervención^(22)^.

En China, un estudio cuasi-experimental observó que el uso de una aplicación móvil mejoró la comprensión y la concientización de los pacientes sobre la insuficiencia cardíaca en relación con las habilidades de autogestión del síndrome^(23)^. En Corea del Sur, un estudio piloto, cuya intervención se basó en una aplicación móvil, dio como resultado una mejora significativa en los puntajes de la escala digital de AS, el conocimiento sobre la enfermedad y los comportamientos de autocuidado en pacientes con IC^(24)^. Otra investigación evidenció una mejora significativa en la calidad del autocuidado tras tres meses de uso de la aplicación móvil^(25)^.

Un metaanálisis destinado a evaluar el impacto de las intervenciones de e-salud en el manejo de la enfermedad en pacientes con IC, que incluyó ensayos clínicos controlados y aleatorizados de diversos países (China, EE. UU., Reino Unido, Suecia, Australia), sugiere que las intervenciones de salud basadas en la tecnología de la información son replicables en diversas otras realidades, con el resultado principal de mejorar el pronóstico de las personas con la enfermedad y la capacidad de reducir la mortalidad y las reingresos, además de favorecer la mejora en la calidad de vida^(26)^.

En el presente estudio, los participantes mostraron una mejora en las puntuaciones de mantenimiento, manejo y confianza en el autocuidado. El marco teórico adoptado postula que los comportamientos de mantenimiento están influenciados por factores externos y que la toma de decisiones puede ocurrir de manera consciente o inconsciente, a partir de la interacción entre las características individuales y el contexto en el que se encuentra la persona^(5)^. Por lo tanto, se deduce que la exposición a la aplicación ejerció una influencia positiva en el desempeño del autocuidado, con potencial para ser incorporada como herramienta de apoyo en las estrategias educativas llevadas a cabo por enfermeros y por el equipo multidisciplinario.

Entre las limitaciones del presente estudio, se destacan el perfil restringido de los participantes con niveles adecuados de AS y la naturaleza multifactorial del autocuidado, influenciado por condiciones impuestas por el propio síndrome, la edad avanzada, el bajo nivel educativo, aspectos psicológicos y el apoyo social insuficiente^(1,5)^. Dichas características limitan la generalización de los hallazgos, los cuales deben interpretarse con cautela. Además, el autoinforme sobre el uso de la aplicación está sujeto al sesgo de deseabilidad social y a la ausencia de métricas objetivas de participación, acceso y tiempo de uso, lo que constituye una limitación adicional que deberá superarse en investigaciones futuras.

Se recomienda realizar ensayos clínicos aleatorios con muestras más amplias, durante un período más prolongado y en diferentes contextos de atención, utilizando la aplicación «Tum-Tum» para ampliar la comparabilidad de los resultados y evaluar la eficacia de la herramienta.

## CONCLUSIÓN

Este estudio identificó un aumento en las puntuaciones de mantenimiento, manejo y confianza en el autocuidado de personas con insuficiencia cardíaca tras el uso de la aplicación móvil «Tum-Tum». Los resultados brindan a los profesionales de la salud, sobre todo a los enfermeros, una herramienta educativa viable, de bajo costo y fácil de manejar para apoyar la práctica asistencial, especialmente las actividades de educación en salud, potenciando las competencias y habilidades para el autocuidado y la toma de decisiones en esta población.

## Data Availability

El conjunto completo de datos que respalda los resultados de este estudio se publicó en el propio artículo.
